# Oncogenic KRAS mutations drive immune suppression through immune-related regulatory network and metabolic reprogramming

**DOI:** 10.1038/s41419-025-08101-1

**Published:** 2025-11-03

**Authors:** Lin Tian, Hui Li, Heran Cui, Chenchen Tang, Peiyan Zhao, Xinyue Wang, Ying Cheng

**Affiliations:** 1 Medical Oncology Translational Research Lab, Jilin Cancer Hospital, Changchun, China; 2 Jilin Provincial Key Laboratory of Molecular Diagnostics for Malignant Tumor, Jilin Cancer Hospital, Changchun, China; 3 Biobank, Jilin Cancer Hospital, Changchun, China; 4 Postdoctoral Research Workstation, Jilin Cancer Hospital, Changchun, China; 5 Department of Thoracic Oncology, Jilin Cancer Hospital, Changchun, China

**Keywords:** Cancer microenvironment, Cancer metabolism, Cancer immunotherapy

## Abstract

The *KRAS* mutation represents the most prevalent oncogenic alteration observed in human cancers. Its primary role involves directly driving malignant tumor development and growth through the activation of downstream signaling pathways. Increasing evidence suggests that KRAS significantly affects the immune response of *KRAS*-mutant tumors by modulating immune-related signaling and inflammatory pathways. In addition to broadly regulating the KRAS-associated immune signaling, KRAS influences immune cell phenotype and function by triggering tumor metabolic pathways. Here, we reviewed the *KRAS* mutation-associated immune microenvironment features and discussed how KRAS remodels the immune microenvironment by regulating immune-related molecules, inflammatory factors, and multiple metabolic pathways, offering insights that could be useful for developing effective immune-responsive therapies for *KRAS*-mutant tumors.

## Facts

- Oncogenic KRAS directly induces immunosuppressive cell infiltration and attenuates immune cell function by inflammatory pathways and immune-related signaling.
- Oncogenic *KRAS* drives the crosstalk between metabolism and tumor immunosuppression to facilitate tumorigenesis.
- Innovative combination therapies that target immune-related molecules and tumor metabolism are emerging as promising strategies to overcome immunotherapy resistance in *KRAS*-driven tumors.

## Open questions

- What are the defining characteristics of immune microenvironment in *KRAS*-driven tumors, and what are the primary mechanisms by which KRAS mutations remodel the immune cell function?
- What are the distinct metabolism features of *KRAS*-driven tumors, and what are the precise mechanisms by which the tumor metabolism drives immune escape in *KRAS*-driven tumors?
- How can novel therapeutic combinations be optimized to enhance the efficacy of immunotherapies or targeted therapies in *KRAS*-mutant tumors?

## Introduction

Somatic mutations leading to activation of oncogenes or functional deficiency of tumor-suppressor gene are known to induce tumorigenesis. Hundreds of driver genes have been identified in cancers that cause abnormal and uncontrolled cell growth. Among these, *RAS* mutations represent the most frequent oncogenic alteration in human cancers [1]. The *RAS* gene family consists of Kirsten rat sarcoma (*KRAS*), Harvey rat sarcoma (*HRAS*), and Neuroblastoma RAS viral oncogene homolog (*NRAS*) genes. RAS is a membrane-bound protein that binds guanosine triphosphate (GTP). In response to the induction of various factors, the *RAS* gene develops mutations and persistently binds GTP, resulting in the hyperactivation of downstream signaling pathways and causing aberrant cell growth, proliferation, and tumorigenesis [2]. Over the years, attempts have been made to inhibit the KRAS signaling pathway to prevent its overactivation at both upstream and downstream points. However, KRAS is not sensitive to the inhibition of upstream growth factor receptor signaling, while targeting downstream effector molecules is hindered by the activation of compensatory resistance mechanisms [3, 4]. In clinical studies, *KRAS*^G12C^ mutation inhibitors, mainly Adagrasib and Sotorasib, have achieved promising outcomes, however, about 12% of patients with *KRAS*^G12C^ mutations exhibit primary resistance, and those who respond to therapy frequently develop acquired resistance [5, 6]. Moreover, developing targeted drugs for other point mutations within the *KRAS* gene is at a bottleneck, with approximately 70% of *KRAS*-mutant Non-small cell lung cancer (NSCLC) patients with non-G12C point mutations still lacking available therapeutic inhibitors [7].

Recent evidence suggests that oncogenic mutations trigger immune escape by modulating the tumor microenvironment (TME) [8, 9]. Oncogenic *KRAS* mutations can alter immune cell phenotype and function through diverse pathways, including modulation of various immunosuppressive molecules, inflammatory factors, chemokines, and signaling pathways [10, 11]. The KRAS-associated inflammatory response involves complex crosstalk that reshapes the immune microenvironment. A KRAS-inflammatory positive feedback loop has been documented in *KRAS*-mutant tumors. For instance, the *KRAS* mutation induces an inflammatory signal, facilitating cytotoxic CD8^+^T cell exhaustion, monocyte differentiation, and the conversion of pro-inflammatory CD4^+^T cells to anti-inflammatory regulatory T cells (Tregs) [12, 13]. Conversely, an increased inflammatory response can increase the *KRAS* mutation frequency, leading to tumorigenesis [14].

An additional key route by which *KRAS* mutations remodel the TME is modulating tumor metabolism. Growing data indicate that altered metabolic features of tumors are inextricably linked to tumorigenesis and immune regulation [15]. Somatic mutations in oncogenes, such as KRAS, significantly modify tumor metabolic processes, which are key factors in inducing tumor progression and immune escape [16]. Oncogenic *KRAS* mutations engage in various metabolism pathways in tumors, including lipometabolism, glycometabolism, amino acid metabolism, and nucleotide metabolism, to meet the high biosynthetic demands of proliferating tumor cells and accelerate their growth [17, 18]. *KRAS* mutations exert a dual influence on the immune microenvironment and subsequent immune response. *KRAS* mutation increase glycolysis metabolism, oxidative phosphorylation, and hypoxia pathway activation, promoting tumor development and restricting CD8^+^PD-1^−^T infiltration, thereby suppressing the anti-tumor immune response [19]. Conversely, KRAS also increased glutamate levels and glutaminase expression. Glutamate abundance facilitates the activation of T cells in response to anti-programmed cell death protein 1(PD-1) [20].

## Immunomodulatory effects of oncogenic KRAS

*KRAS*-mutant TME exhibits significant immunosuppression, characterized by CD8^+^T cell exhaustion, suppressive monocyte and macrophage subsets, and an increased abundance of Tregs [21]. These immunosuppressive cells reshape the TME and affect the fate of the tumor cells by prompting further mutations and accelerating immune evasion.

**Fig. 1:**
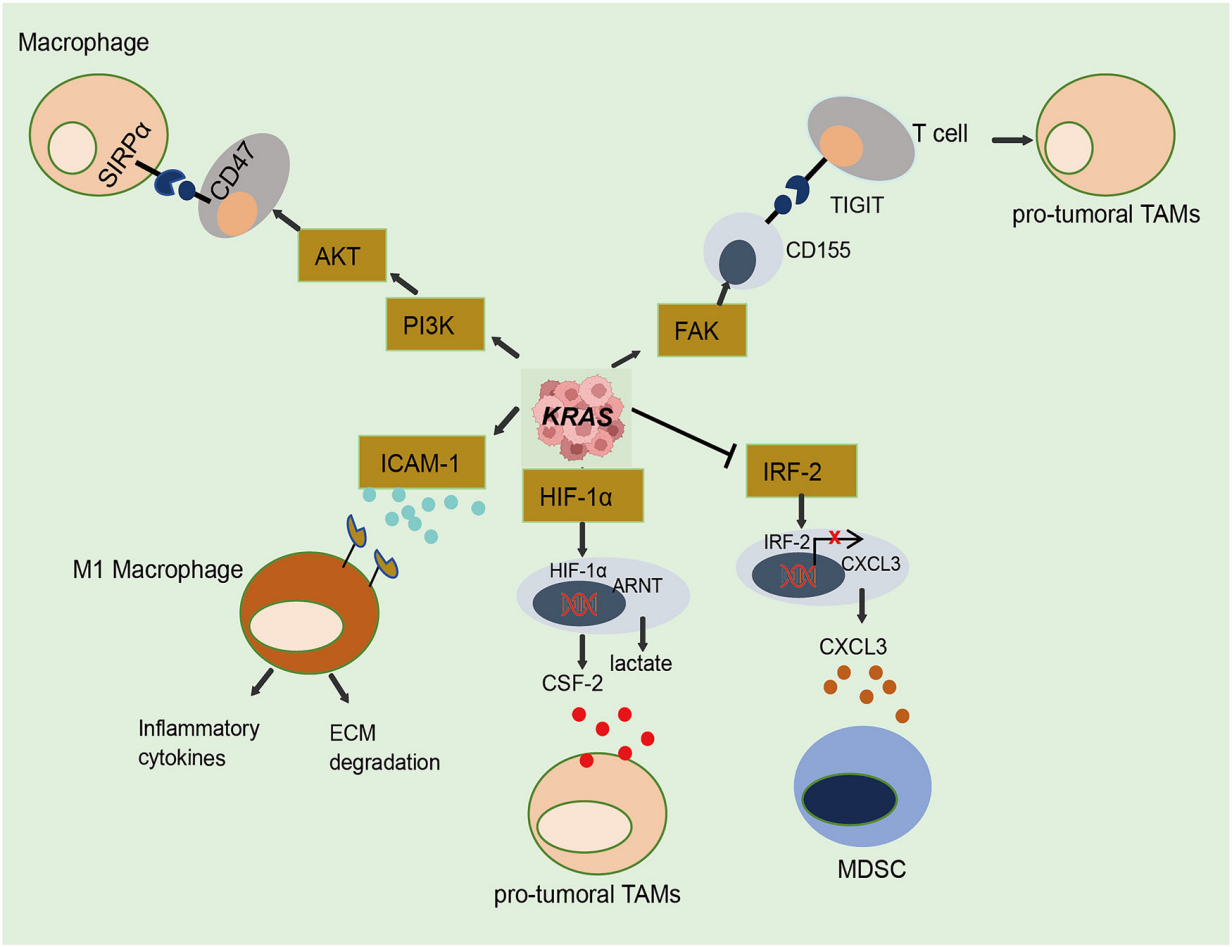
Effects of KRAS on the phenotype and function of TAMs. Oncogenic KRAS facilitates the pro-tumor activity of TAMs by eliciting the production of various immunosuppressive molecules, inflammatory factors, chemokines, and signaling pathways. KRAS drives the expression and secretion of ICAM-1, CSF-2, and CXCL3 to recruit TAMs and regulate the immune function. KRAS accelerates immune evasion by regulating macrophage-associated immune checkpoint expression such as CD47 and CD155.

Circulating monocytes and tumor-infiltrating macrophages play a key role in stabilizing immunosuppressive state of *KRAS*-mutant TME [22, 23], as illustrated in Fig. 1. KRAS dampens the antitumor activity of tumor-associated macrophages (TAMs) by eliciting the production of various immunosuppressive molecules, inflammatory factors, chemokines, and signaling pathways. *KRAS*^G12D^ in pancreatic acinar cells recruits TAMs via upregulation the expression of intercellular cell adhesion molecule-1 (ICAM-1), accelerating the formation of precancerous lesions. Subsequently, infiltrating macrophages release matrix-degrading enzymes, including matrix metalloproteinase9, and cytokines, such as tumor necrosis factor (TNF), which collectively drive *KRAS*^G12D^-caused acinar cell metaplasia. Notably, the use of ICAM-1 neutralizing antibodies has been shown to block macrophage infiltration, thus decreasing the *KRAS*^G12D^-caused formation of precancerous lesions [24]. KRAS promotes the production of tumor-originating colony stimulating factor 2 and lactate by enhancing hypoxia-inducible factor-1α (HIF-1α) stabilization. In this regard, *KRAS*^G12V^ activation increases the production of reactive oxygen species, which inhibit the activity of prolyl hydroxylase, hence decreasing the hydroxylation of HIF-1α and enhancing its stability [25]. Chemokines are essential cytokines for infiltrating peripheral blood-derived monocytes into the interior of tumor tissues, and KRAS preferentially regulates macrophages through modulating chemokine secretion. KRAS^G12D^-expressing tumors can recruit myeloid-derived suppressor cells (MDSCs) in the TME through the C-X-C motif chemokine ligand 3 (CXCL3)/C-X-C motif chemokine receptor 2 (CXCR2) axis, which is mediated by interferon regulatory factor 2 (IRF2) and increases MDSCs migration into the TME. Enhancing IRF2 expression or silencing CXCR2 expression can reverse anti-PD-1 resistance in KRAS-expressing tumors [26]. In addition to recruiting TAMs via chemokines, KRAS can evoke the immunosuppressive function of macrophages by inducing immune checkpoint signaling. CD47, as an anti-phagocytic signal, facilitates the reduction of macrophage phagocytosis, which is regulated by oncogenic *KRAS*^G12C^ in lung adenocarcinoma (AD). KRAS was found to suppress miR-34a expression by activating PI3K/STAT3 signaling, alleviating the post-transcriptional depression of CD47 by miR-34a [9].

**Fig. 2:**
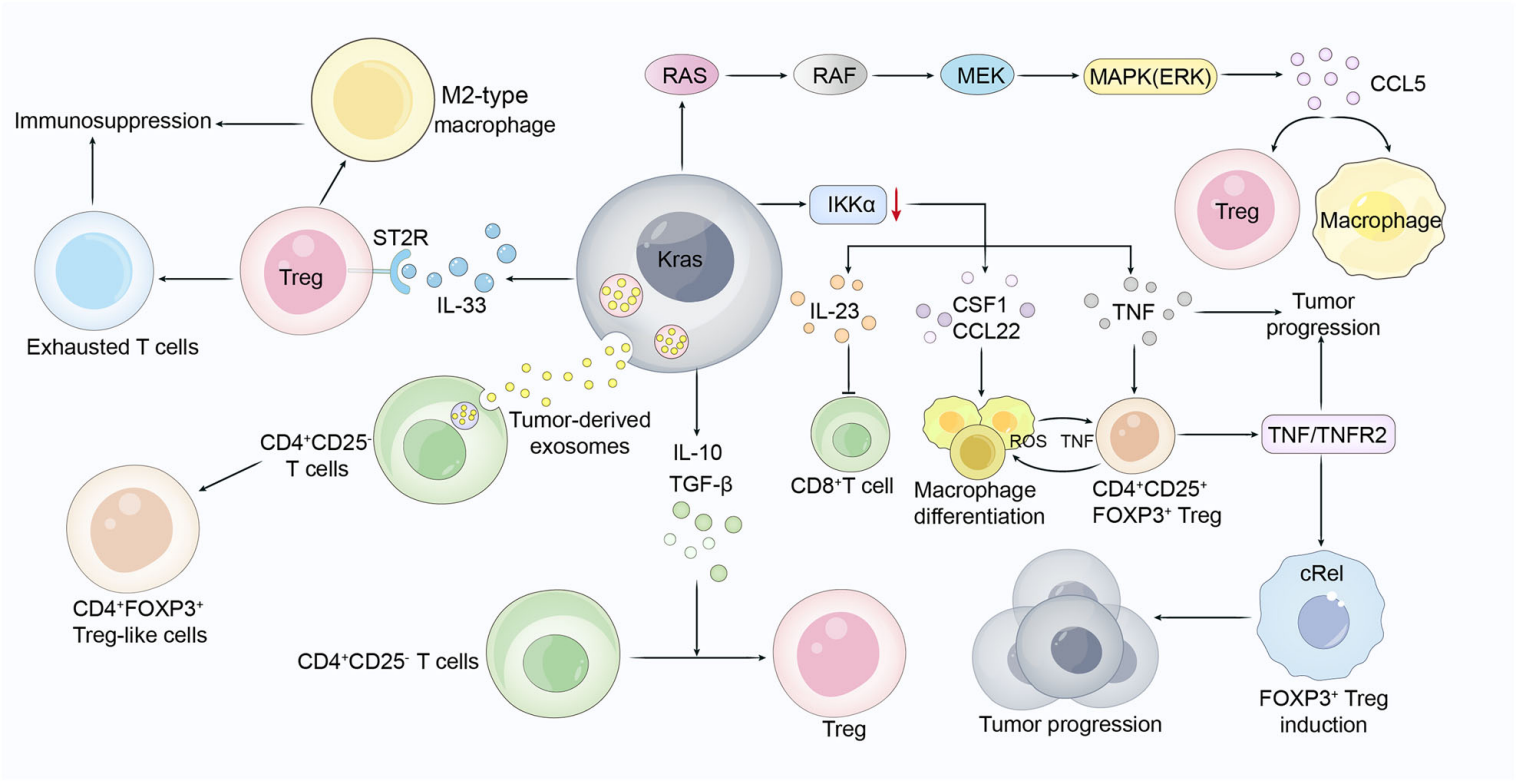
KRAS regulates the pro-tumoral functions of Treg cells. KRAS promotes differentiation of pro-tumorigenic Treg cells by regulating the expression of cytokines such as IL-33, IL-10, TGF-β and IL-23. KRAS also converts CD4^+^CD25^-^T cells into Tregs by upregulating the levels of IL-10 and TGF-β.

Tregs also show significant immunomodulatory effects in *Kras*-mutant tumors (Fig. 2). In *KRAS*^G12D^-initiated lung AD, decreased IκB kinase α (IKKα) expression was found to enhance differentiation of pro-tumorigenic Treg cells. IKKα deficiency induced the expression of the cytokines CSF1, CCL22, TNF, and IL-23 through the TNF/TNFR2/cRel signaling cascade, promoting Treg cell generation [27]. Among these cytokines, TNF could induce the CD4^+^CD25^+^FoxP3^+^Treg differentiation, CSF1 and CCL22 drive the differentiation of F4/80^+^CD11b^+^ monocytes to macrophages, and IL-23 inhibited CD8^+^T cell infiltration and promoted inflammation and tumor incidence [28, 29]. KRAS can also influence the immune microenvironment by inducing a shift from T cells to Treg phenotypes. The tumor-derived exosomes from the *KRAS-*mutant NSCLC patients have been found to transform naïve CD4^+^CD25^−^T cells to CD4^+^FoxP3^+^ Treg-like cells, a process independent of cytokine signaling (Fig. 2). However, *KRAS* mutations in tumors did not increase the proliferation of true FoxP3^+^Tregs, which could be attributed to the converted Treg-like population originating from pre-existing Treg-like cells in the CD4^+^ population rather than from FoxP3^−^T cell conversion in *KRAS* mutant tumors [30]. Another study further revealed that *KRAS*^G12D^ in tumors could convert the CD4^+^CD25^−^T cells into Tregs by up-regulating the levels of interleukin-10 (IL-10) and transforming growth factor-β (TGF-β) [31, 32].

T cell infiltration and activation are significantly suppressed in *KRAS*-mutant tumors. *KRAS* mutant patients typically exhibit lower cytotoxic CD8^+^T cells infiltration compared to patients with wild-type KRAS. The fate of T cells is influenced by various immune cells and factors, including cancer-associated fibroblasts (CAF), macrophages and Tregs. CAF-derived IL-33 inhibits infiltration and promotes exclusion of CD8^+^T-cell, which is linked to increased neutrophil infiltration via CXCL1-CXCR2 signaling [33]. PDAC cell lines generated from the KPC (*Kras*^LSL-G12D/+^; *Trp53*^fl/fl^; *Ptf1a*^Cre/+^) mouse model showed high levels of transglutaminase 2 (TGM2) expression, which regulates vesicle trafficking by modulating microtubule network density and dynamics and promotes the secretion of immunosuppressive cytokines such as G-CSF and GM-CSF, thus impairing T cell activation [34]. In the KMF (KRAS, MYC, Focal adhesion kinase (FAK)) synthetic ovarian tumor model, FAK activation regulates the expression of CD155, a checkpoint ligand for T cell immunoreceptor with immunoglobulin and immunoreceptor tyrosine-based inhibitory motif domains (TIGIT). FAK inhibitors, in combination with anti-TIGIT, lowered the PD-1 and TIGIT levels on infiltrating CD4^+^CD8^+^T cells, as well as the abundance of granulocytes, monocytes, and macrophages [27]. Moreover, the cytokine IL-33 secreted by *KRAS*^G12D^-mutant lung cancers induces the expression of suppression of tumorigenicity 2 (an IL-33 receptor) in Tregs, leading to the exhaustion of CD8^+^ cytotoxic T, NK, and Th1 cells, and M2-type macrophage differentiation [35].

## Cancer-specific neoantigens of *KRAS*-mutant tumors

*KRAS* mutations can initiate multi-mechanism immune escape events involving T cell-mediated immunotherapy. Cancer-specific neoantigens induced by somatic mutations are critical immune modulators. Neoantigens presented by class I or II human leukocyte antigens (HLA) can trigger antitumor responses of cytotoxic T cells via alpha-beta T cell receptors (αβTCR). Several *KRAS* mutation-specific peptides presented by HLA have been identified as targets in TCR-dependent immunotherapy [36, 37]. The peptides from *KRAS* mutation status (G12V and Q61H/L/R) can be presented by HLA-A3 and HLA-A1, respectively, and recognized by TCR [38]. Besides, HLA-peptide prediction algorithms have revealed that HLA-A*11:01 may bind peptides harboring *KRAS* mutations in patients with various common cancer types. The transfer of KRAS-reactive TCR-engineered T cells that recognize multiple HLA-A*11:01^+^ tumors has been shown to significantly induce tumor regression in immunized HLA-A*11:01 transgenic mice [39]. Poole et al. also demonstrated that in human cancers, the *KRAS*^G12D^ decamer peptide can be presented in the context of HLA-A*11 (HLA-A*11-*KRAS*^G12D^) [40]. Impairment of the presentation of new antigens due to loss of HLA can facilitate immune evasion. Loss of heterozygosity (LOH) in HLA is an immune evasion mechanism that arises under intense selective stress from the microenvironment during advanced stages of tumor evolution [41]. A case report described a direct mechanism of immune evasion in tumors expressing mutant *KRAS*^G12D^. A copy-neutral LOH on chromosome 6 encoding the HLA-C*08:02 class I major histocompatibility complex molecule was found to facilitate tumor evasion from T-cell transfer therapy. This finding is particularly significant given that HLA-C*08:02-restricted tumor-infiltrating lymphocytes consisting of four unique T-cell clonotypes, could specifically target *KRAS*^G12D^, suggesting that treatment with CD8^+^T cells could lead to regression of lung metastases with metastatic colorectal cancer expressing both mutant *KRA*S^G12D^ and HLA-C*08:02 [42].

## Crosstalk between KRAS and T-cell based immunosuppressive molecules

Clinical observations have revealed that PD-L1 gene alterations in the context of KRAS mutations lead to in an inflammatory TME and tumor immunogenicity [43]. *KRAS* mutation-induced PD-L1 up-regulation triggers CD3^+^T cell apoptosis via the PD-1/PD-L1 axis [44]. Direct regulation of PD-L1 expression by KRAS has been reported to be dependent on the MEK-ERK pathway [45]. FOS-related antigen 1 (FRA1), a MEK/ERK-dependent oncogenic transcription factor, induced the expression of PD-L1 in the *KRAS* mutated cells. Inhibition of ERK activation or FRA1 silencing can reduce the PD-L1 expression. PD-L1 was positively regulated by KRAS via p-ERK but not p-AKT signaling. Another study found that oncogenic RAS signaling increased PD-L1 mRNA levels and was associated with the AU-rich element-binding protein tristetraprolin (TTP). MEK signaling, downstream of RAS, controlled TTP activity through the kinase MK2, while TTP negatively regulated PD-L1 expression via AU-rich elements in the 3′ UTR of PD-L1 mRNA [46]. Conversely, several studies have found a dissenting perspective on the association between KRAS and PD-L1. Lastwika and colleagues reported that PD-L1 expression depended on active PI3K-AKT-mTOR signaling, independent of the driving oncogene driver (*EGFR*, *KRAS*) or exogenous cytokine stimulus [47]. It is expected that distinct *KRAS* oncogene states may activate different downstream signaling pathways that regulate PD-L1 levels. For instance, the *KRAS*^G12D^ point mutation suppressed PD-L1 expression through the P70S6K/PI3K/AKT axis [48]. In *KRAS*^G12C^-mutant NSCLC, PD-L1 levels were positively correlated with KRAS [49]. In *KRAS*^G12V^-mutant NSCLC, the oncogenic driver increased PD-L1 expression via the TGF-β/EMT pathway [50].

Protein tyrosine phosphatase non-receptor type 11 (PTPN11 or SHP2) and PD-1-SHP2 signaling suppresses T cell activation by restraining myelocyte differentiation [51]. SHP2 plays a pivotal role in regulating both tumor and immune cells through its interaction with its substrates [52]. SHP2 in tumor cells promotes tumor formation and progression via the RTK-RAS-ERK signaling pathway [53] and facilitates immune evasion by up-regulating PD-L1 expression. In immune cells, SHP2 can dampen CD8^+^T cell cytotoxicity and macrophage phagocytosis. Tang et al. demonstrated that SHP2i treatment alone could increase antitumor immune responses of T cells through the SHP2/RAS/ERK pathway, as well as recruit S100A8^hi^ gMDSCs, which suppress the cytotoxic function of Klrg1^+^ CD8^+^ effector T cells depending on the CXCR2 ligand expression in *KRAS*^G12C^-mutant NSCLC. The combination of SHP2i and CXCR2i could increase the Klrg1^+^ CD8^+^ T cell infiltration while blocking gMDSC immigration, improving survival in *KRAS*-mutant models [54].

## Modulation of the immune microenvironment by co-mutations with KRAS and different KRAS subtypes

*KRAS*-mutant tumors exhibit biological differences, heterogeneous characteristics of the immune microenvironment characteristics, and distinct responses to treatments, which may be associated with co-mutations in other genes (TP53, STK11/LKB1, MYC, KEAP1, and CDKN2A/CDKN2B) [55–58]. Among these, *TP53* and *LKB1* are two genes that are frequently co-mutated with *KRAS*. Tumors with *KRAS*/*TP53* co-mutations highly express PD-L1 and induce cytotoxic T-cell infiltration [59, 60]. Conversely, an increasing body of evidence suggests that tumors with *KRAS*/*LKB1* co-mutations are usually negative for PD-L1 expression, accompanied by sparse tumoricidal immune infiltrates [61–65]. PD-L1 expression in the *KRAS*/*LKB1*/*TP53* triple mutation showed no difference compared to the *KRAS*/*LKB1* co-mutation group [66, 67]. Single-cell RNA sequencing revealed that *KRAS*/*TP53*-driven cancer cells contribute to T cell dysfunction as well as pro-tumorigenic behaviors of macrophages, as evidenced by the infiltration of immunosuppressive subtypes of T cells (LAG3^+^CD8^+^T and LAG3^+^CTLA4^+^CD8^+^T) and enrichment of FOLR2^+^LYVE1^+^ macrophage subtypes which are associated with poor prognosis [68]. Immune profiles of *KRAS*/*TP53* co-mutations were characterized by significant enrichment of pattern recognition receptors for antigen recognition, antigen presentation, dendritic cell maturation, and immune response [61]. Accordingly, *KRAS*/*TP53* co-mutations are more likely to benefit from immunotherapy, presenting as a ‘hot’ (immune-inflamed) phenotype compared with *KRAS* mutant tumors, as shown in Table 1. In *KRAS*/*LKB1*-mutant lung AD, CD3^+^/CD8^+^T subsets, CD68^+^macrophages and mature DCs are significantly decreased, immune co-stimulatory molecules, immune checkpoint-related molecules and type I IFN signaling signatures showed significantly downregulated expression, in which tumors showed insignificant response to ICB. Lung cancer cells with *KRAS*/*MYC* cooperation exhibited more pronounced proliferation and aggressiveness, as well as suppressive and inflammatory features of the immune microenvironment, compared to *KRAS*^G12D^ mutations alone [69, 70]. *KRAS*^G12D^-driven lung tumors are dependent on Myc activity to maintain proliferation and immunosuppression. Myc induced high expression of CCL9 and IL-23 in epithelial cells to reprogram inflammation and immune suppression. CCL9 mediates the dysfunction of PD-L1-dependent T and B cells and macrophage infiltration. Moreover, IL-23 contributes to the removal of adaptive T and B cells and innate immune NK cells [70]. The combined actions of Myc and KRAS could inhibit functional immune cell infiltration by suppressing the type I IFN pathway. Restoring IFN signaling promoted NK and B cell infiltration and remodeled the immune microenvironment [71]. In the combination of *lsl-KRAS*^*G12D*^ and one copy of *Rosa26* ^*DM-lsl-MYC*^ (KMC) mouse model, Myc-interacting zinc finger protein (Miz1) was found to be involved in Myc-dependent transcriptional repression. The Myc/Miz1 transcriptional repressor complex could suppress the expression of promoters of type I interferon regulators IRF5, IRF7, STAT1, and STAT2 and consequently inhibited the interferon-related B and NK cell-mediated immune surveillance [72].

**Table 1:** Modulation of the immune microenvironment by co-mutations with KRAS.

Groups	KRAS/LKB1	KRAS/TP53	KRAS/MYC	KRAS/LKB1/TP53 tri-mutation
Characteristics				
Mutation frequency	8–31% [60, 61]	31–46% [60, 61]	rare	11–19% [63, 64]
Percentage of PD-L1-positive tumors	10% [62]	56.3% [62]	_	25% [62]
PD-L1/TIL paradigm	Negative/Negative [60]	Positive/Positive [60]	Positive/Positive [60]	Positive/Negative [60]
immune cell populations	1. Few tumoricidal immune infiltrates	1. T cell dysfunction and pro-tumoral macrophages [68]	1. Suppressive and inflammatory features of the immune infiltrates [71, 72]	1. T-cell exhaustion [63, 67]
	2. Low cytotoxicity of CD4^+^T and CD8^+^ T [64]	2. T-cell-driven cytotoxicity [61]	2. Dysfunction of T, B cells and macrophages [71, 72]	2. Increased amounts of neutrophils with immune-suppressive function [64]
	3. Few CD8^+^T, CD68^+^macrophages and matured DC [65]	3. Infiltration of immunosuppressive T cells (LAG3^+^CD8^+^T and LAG3^+^ CTLA4^+^ CD8^+^T) [68]		
		4. FOLR2^+^ LYVE1^+^macrophage subtypes [68]		
Immune-related molecules	1. PD-1, CTLA-4, TIM-3 and LAG-3 downregulation in tumors [61, 67]	1. PD-1, CTLA-4, TIM-3 and LAG-3 upregulation [61, 67]	1. CCL9 and IL-23 in epithelial cells to reprogram inflammation and immune suppression [70]	1. PD-L1 upregulation [63]
	2. low PD-L1 expression [64]	2. CCL-5, CXCL-9, CXCL-10, CXCL-11 and CXCL-13 upregulation [65]	2. type I IFN pathway downregulation [71]	2. CD28, CD86, CTLA-4, TIM-3 and HLA-DR downregulation reduced amounts of T cells in tumors [67]
	3. Low production of IFN-γ by CD4 + /CD8 + T [64]	3. HLA-DR, CD28, ICOS, CD80 and CD86 upregulation [61, 67]	3. IRF5, IRF7, STAT1, and STAT2 inhibits the interferon-related B and NK cell-mediated immune surveillance [72]	3. CXCL-7, G-CSF and IL-6 upregulation - increased amounts of immune-suppressive neutrophils [64]
	4. HLA-DR, CD28, ICOS, CD80 and CD86 downregulation [61]	4. Presentation of antigen recognition and presentation [62, 67]		
ORR to ICI (NSCLC)	≤10% [62]	≥30% [62]	_	_

*KRAS* kirsten rat sarcoma viral oncogene, *LKB1* liver kinase B1, *TP53* tumor protein P53, *PD-L1* programmed death-1, *TIL* tumor infiltrating lymphocyte, *LAG3* lymphocyte activation gene 3, *CTLA4* cytotoxic T-lymphocyte-associated antigen 4, *FOLR2* folate receptor 2, *LYVE1* recombinant lymphatic vessel endothelial hyaluronan receptor 1, *PD-1* programmed cell death protein 1, *TIM-3* T cell immunoglobulin and mucin domain-containing protein 3, *IFN-γ* interferon-gamma, *HLA-DR* human leukocyte antigen DR locus, *STING* stimulator of interferon genes, *CCL-5* CC chemokine ligand 5, *CXCL* C-X-C motif ligand, *IL* Interleukin, *IRF* interferon regulatory factor, *STAT* signal transducer and activator of transcription, *NK* natural killer cell, *G-CSF* granulocyte colony-stimulating factor, *ORR* objective response rate, *ICI* immune checkpoint inhibitors, *NSCLC* non-small cell lung cancer.

KRAS mutations have been extensively documented in multiple human cancers, with mutation rates varying significantly across different cancer types. Analysis of a cohort of 10,820 patients revealed that pancreatic cancer exhibited the highest prevalence of *KRAS* mutations (73.51%), followed by colorectal cancer (41.45%), and lung cancer (11.24%). The *KRAS*^G12D^ mutation is the most common in pancreatic cancer, colorectal, and gastric cancer. Besides, the *KRAS*^G12V^ mutation exhibits a relatively high mutation frequency in uterine cancer, while the *KRAS*^G12C^ mutation is most frequently observed in lung cancer [73]. The TME of KRAS-mutated tumors exhibits diverse compositions and function of immune cells and subtype-specific immune suppression, leading to differences in immune therapy responses. Ji et al. evaluated the impact of *KRAS*^G12D/G12V^ mutations on the TME by analyzing a larger scale exome data from the TCGA dataset in pancreatic cancer. Compared with *KRAS*^G12D^ mutations, *KRAS*^G12V^ mutations were associated with significantly increased T cell infiltration, a higher proportion of CD4^+^T memory cells, monocytes, DC, and mast cells, and reduced inhibitory cytokine secretion [74]. In lung cancer, the G12D mutation tends to trigger immune escape by modulating the downstream PI3K/AKT pathway and diminishing the activation of the HMGA2-CXCL10/11 signaling pathway, thereby reducing the expression of PD-L1 and the infiltration of tumor-infiltrating lymphocytes (TILs) [48]. Su et al. analyzed a cohort of 103 patients harboring different KRAS mutation subtypes: G12A, G12C, G12D, and G12V, and evaluated differences in immunotherapy benefits among different KRAS subtypes in lung cancer. The G12A and G12V subtypes were more enriched in interferon-related responses, with G12V exhibiting greater abundance in inflammatory pathway and TNF-α signaling mediated by NF-κB [75]. In view of the variations in the characteristics of the immune microenvironment among different KRAS subtypes, KRAS subtypes could serve as potential biomarkers for predicting the efficacy of immune checkpoint inhibitors.

## Inflammatory response in *KRAS*-mutant tumors

**Fig. 3:**
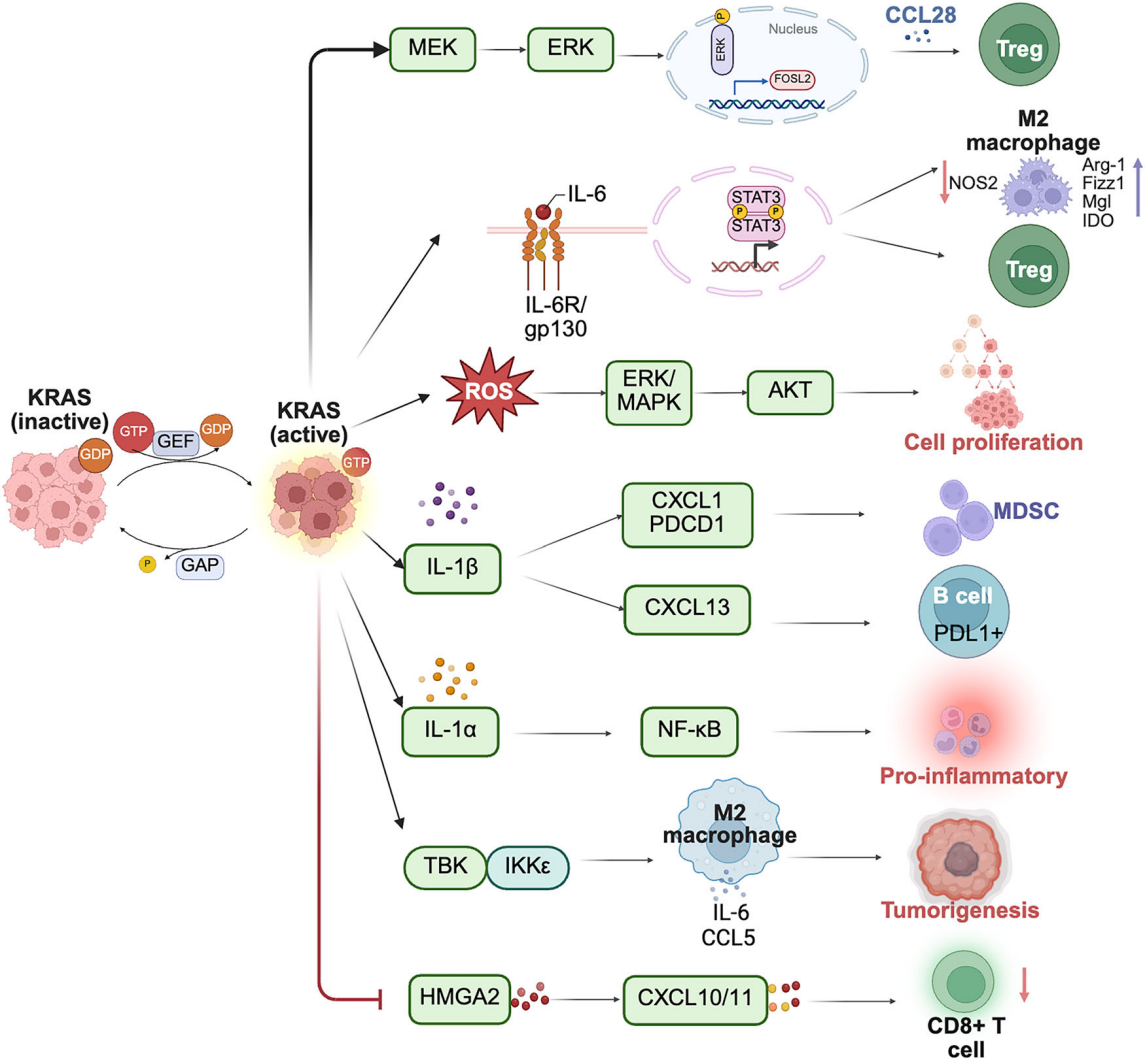
KRAS-induced inflammation in cancer. KRAS mutations are involved in the activation of ERK/MAPK and NF-κB pathways and promote the transcription of various cytokines and chemokines, such as IL-1α, IL-1β, IL-6, and CCL5. The oncogenic *KRAS* mutation involved in the inflammatory response contributes significantly to the tumor progression network and remodels the immune microenvironment.

Oncogenic *KRAS* mutations are strongly associated with pro-tumoral inflammation. As an anti-inflammatory modulator, KRAS can reshape the TME by secreting inflammatory cytokines and chemokines. The *KRAS* mutation induces the NF-κB pathway, promoting the transcription of several cytokines and chemokines [76, 77]. This aberrant signaling facilitates cytotoxic CD8^+^T cell exhaustion and monocyte differentiation and converts pro-inflammatory CD4^+^T cells to anti-inflammatory Tregs. Increased inflammatory action can also induce *KRAS* mutation frequency, thereby creating a vicious feedback loop of inflammation [78]. The cytokines transcribed by NF-κB signaling, such as the IL-1 family of proteins, significantly contribute to the tumor progression network and an immunosuppressive phenotype. IL-1β secretion facilitates the infiltration of immunosuppressive myeloid-derived cells and induces the expression of CXCL1 and PD-1 in patients with *KRAS* mutation [79, 80]. IL-6, a pleiotropic pro-inflammatory cytokine, plays a key role in tumor-promoting inflammation, tumorigenesis, and immune modulation in somatic mutant tumor cells [81, 82]. The IL-6 family cytokine receptor gp130, the key driver of “immune-protective” IL-6/gp130/Stat3 classical signaling pathway, was found to accelerate carcinogenesis. *KRAS*^G12D^ lung AD with gp130^F/F^ inhibited suppressor of cytokine signaling (Socs)3-mediated downmodulation of IL-6/Stat3 signaling, enhancing lung cellular proliferation [83]. The IL-6/Stat3/Socs3 is also essential for promoting pancreatic intraepithelial neoplasia (PanINs) progression and the development of PDAC induced by oncogenic *KRAS*^G12D^ mutations. Homozygous deletion of the *Socs3* in the pancreas which initiates aberrant activation of Stat3 accelerates PanIN progression and PDAC development [84]. IL-6 promotes the infiltration of immunosuppressive cells by inducing the expression of pro-tumor type 2 molecules (Arginase 1 and Fizz 1) on macrophages. It also recruits MDSCs by upregulating CXCL1 and IL-17 expression and pro-tumor Treg/T helper 17 cell responses via IL-6/STAT3 signaling induced by the KRAS oncogene [85]. Blocking IL-6 can significantly reduce tumor burden, which is associated with immune microenvironment remodeling and is characterized by cytotoxic CD8^+^T cell expansion and MDSCs reduction [80], as shown in Fig. 3.

Several chemokines have been implicated in the development of inflammation-induced oncogenesis and immunosuppression. CCL5, which is expressed in the epithelial, tumor, and immune cells, contributes to cell migration and chemotaxis. TANK-binding kinase 1 (TBK1) is essential for oncogenic KRAS-induced cell transformation and maintains the survival of KRAS-dependent cancer cells. IKK-related kinases TBK1 and IKKε promote the activation of CCL5 and IL-6. Autocrine CCL5 and IL-6 promote *KRAS*^G12V^-dependent lung cancer transformation [86]. CCL5 secreted by mesenchymal stem cells has also been found to promote the invasion and metastasis of RAS-expressing breast cancer cells [87]. The *KRAS*^G12D^ mutation was found to be negatively correlated with the secretion of CXCL10/CXCL11, a relationship associated with high mobility group protein A2. The decreased secretion of CXCL10/CXCL11 impairs the efficacy of PD-L1 therapy by reducing CD8^+^T cell infiltration. Conversely, stimulating CXCL10/CXCL11 secretion enhances the benefit from ICI therapy for patients with *KRAS*^G12D^-mutant NSCLC [48]. Analysis of the epigenetic landscape revealed that Fos-like antigen 2, a downstream target of the KRAS/MAPK pathway, recruited Tregs by transcriptionally activating CCL28. Accordingly, targeting CCL28 to prevent Treg cell recruitment may be an effective strategy to promote tumor immunotherapy [88].

## Metabolic programming and immune response in the *KRAS*-mutant TME

A growing number of studies have focused on the relationship between metabolic alteration and tumor development or immune regulation in *KRAS*-mutant tumors [17, 89]. Oncogenic *KRAS* mutations actively participate in multiple metabolic processes, including lipometabolism, glycometabolism, amino acid metabolism, and nucleotide metabolism, to meet the high biosynthetic demands and accelerate tumor cell proliferation. Besides, the accumulation or consumption of specific metabolites in the TME drives more somatic mutations, which exacerbates tumor growth and suppresses the immune response.

## Lipid metabolism in the *KRAS*-mutant TME

Lipid metabolism reprogramming in cancer represents a cross-link between the tumor-oncogenic signaling pathways and the function of immune-associated cells within the TME. The TME is primarily characterized by hypoxia, acidity, and nutrient deficiencies, conditions that cause cancer and immune cells to preferentially uptake more lipids for energy storage, biofilm formation, and signaling molecule production. Due to the complex processes of lipid uptake and synthesis, coupled with tumor heterogeneity, immune cells exhibit varied responses to altered lipid metabolism, leading to either anti-tumor or pro-tumor outcomes [90]. While fatty acids and cholesterol, among other factors, can serve as fuel supply that enhances the cytotoxic immune response of T cells or promotes the transition of T cells to memory T cells in specific hypoglycemic and hypoxic cancers, the pro-tumorigenic effects of lipid metabolism continue to be emphasized in the immune microenvironment enriched with adipocytes [91]. For instance, in breast cancers with abundant adipocytes, an activated STAT3 pathway promoting fatty acid oxidation (FAO) induces the immunosuppressive function of CD8^+^T effector cells. The JAK-STAT3 axis activates the FAO pathway during tumor progression, accompanied by inhibition of glycolysis and IFN-γ expression in CD8^+^T effector cells. Suppression of STAT3 expression in CD8^+^T cells blocks FAO signaling and promotes T-cell killing functions, encompassing the expression and secretion of molecules such as IFN-γ, GzmB, and CD107a [92].

**Fig. 4:**
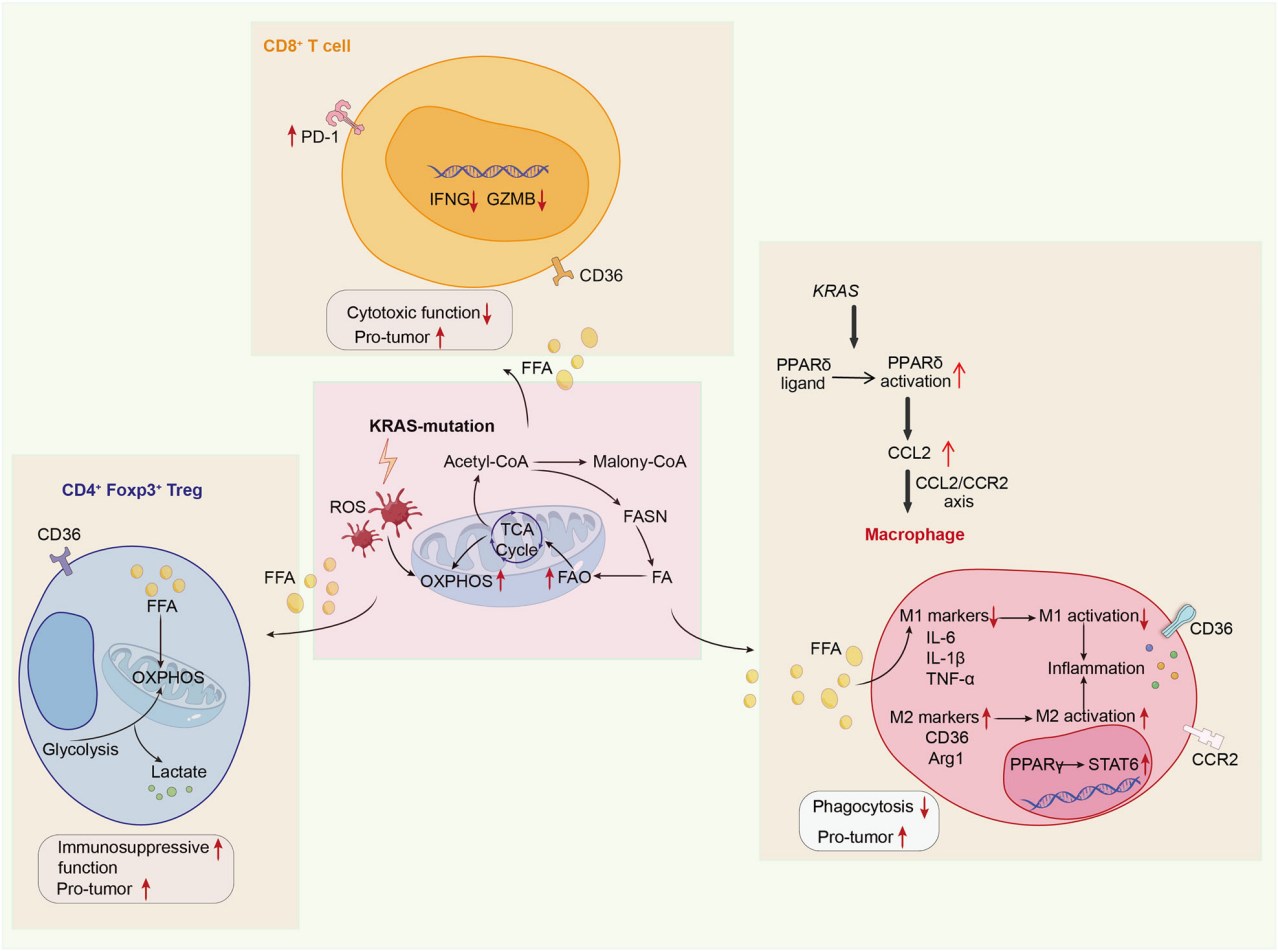
KRAS affects the TME by regulating lipid metabolism. KRAS regulates the expression of genes involved in lipid metabolism, which significantly increases the accumulation of lipid metabolites in tumors. This accumulation impairs the T cell-mediated cytotoxicity, accelerates the pro-tumorigenic ability of CD4^+^FoxP3^+^ Treg cells, and promotes the secretion of CCL2 through the PPARδ activation. CCL2 recruits M2 macrophages, fostering an immunosuppressive TME.

KRAS affects tumor cell development via various lipid metabolism pathways. Genomics and lipidomic analysis have identified that de novo sphingolipid synthesis is an essential pathway for immunosuppression in *KRAS*-driven cancers. Glycosphingolipids were found to induce to down-regulation of IFNγ receptor subunit 1 (IFNGR1), which reduced IFNγ sensitivity and protected cancer cells from immune surveillance by NK and CD8^+^T cells [93]. *KRAS*^G12D^ mutation induced a mutant-specific lipid profile characterized by accumulated phosphatidylcholines and triacylglycerides, as well as sphingomyelins and phosphatidylethanolamine, and a decrease in lysophosphatidylcholines, depending on fatty acid synthase (FASN) overexpression [94]. FASN activated by ERK alters lipid signatures and accelerates the proliferation of KRAS-positive lung cancer cells [95]. Further studies have revealed that FASN inhibitors could accelerate the ROS-and iron-dependent cell death, depletion of lipid droplets, and inhibit de novo lipogenesis, causing FA synthesis-dependent cell death in KRAS-positive tumor cells [94, 96]. Fatty acids could facilitate mutant *KRAS*^G12D^-initiated pancreatic tumorigenesis through a natural ligand to activate peroxisome proliferation-activated receptor δ (PPARδ), a lipid nuclear receptor. Hyperactivation of PPARδ facilitated the secretion of CCL2, which promoted the pro-tumorigenic properties of macrophages and recruited MDSCs into the pancreas via the CCL2/CCR2 axis, thus orchestrating an immunosuppressive TME and promoting *KRAS*-induced pancreatic tumorigenesis in the presence of fatty acid accumulation [97], as shown in Fig. 4. Cholesterol metabolism has also been found to drive regulatory B cell anti-inflammatory function by modulating IL-10 expression. The synthesis of the metabolic intermediate geranylgeranyl pyrophosphate (GGPP) represents a critical regulator of IL-10 production [98]. GGPP plays a key role in mediating cholesterol metabolism and inflammatory responses. In *KRAS*-mutant colorectal cancer (CRC), de novo cholesterol biosynthesis induced GGPP biosynthesis-dependent proliferation of APC/KRAS-mutant CRCs via the GGPP-KRAS/MEK/ERK axis [99].

## Glucose metabolism in the *KRAS*-mutant TME

Glucose metabolism has been established as the primary fuel for tumor proliferation, including glycolysis, pentose phosphate pathway (PPP), and serine synthesis pathway in the cytoplasm, and the tricarboxylic acid (TCA) cycle in the mitochondria. Augmented aerobic glycolysis promotes cancer cell anabolism by increasing carbon intermediates for the biosynthesis of lipids, nucleotides, and amino acids [100]. Tumors consume glucose in the presence of oxygen to produce lactic acid. Proliferating malignant cells maintain their development using aerobic glycolysis, and immune and stromal cells in the TME likewise competitively harness aerobic glycolysis to support their biosynthesis [101]. Lactic acid uptake has been documented in Treg cells via monocarboxylate transporter 1(MCT1) in the glucose-deficient TME caused by competitive consumption by tumor cells, which enhances PD-1 expression. In a high-glucose TME, Tregs were more likely than effector T cells to use glucose metabolism to overexpress PD-1 [102]. Competitive utilization of aerobic glycolysis by tumor cells leads to limited glucose availability for T cells, suppressing CD4^+^T cell-mediated immunosurveillance and facilitating CD8^+^T cell exhaustion [103]. Interestingly, glucose consumption may not be an essential nutrient absorption and regulatory process for tumor cell proliferation; however, tumor cells scramble the glucose and store the glycogen to prevent T cells from utilizing glucose, thus impairing the antitumor immune response [104].

**Fig. 5:**
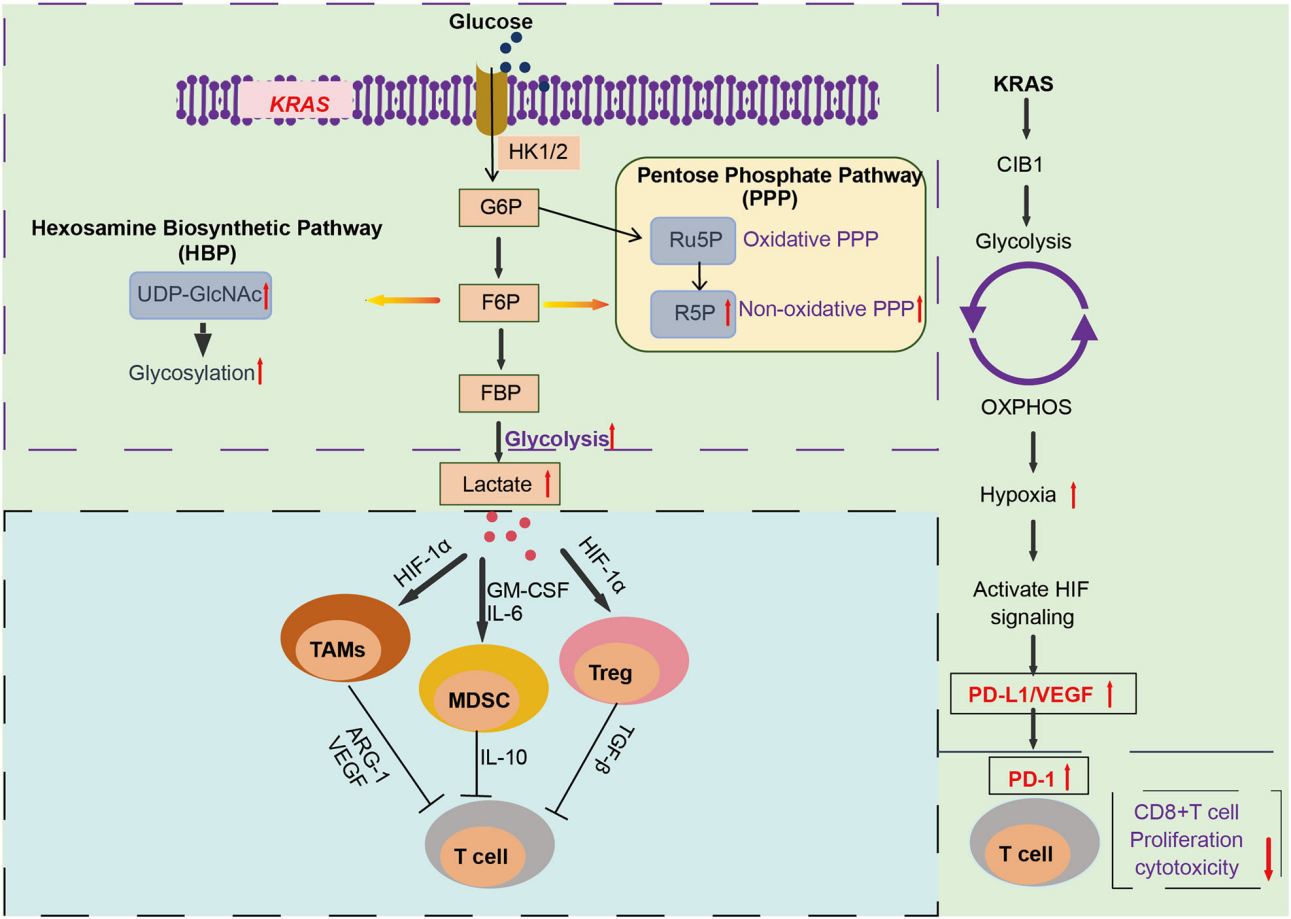
KRAS affects the TME by modulating glucose metabolism. KRAS upregulates HK1/2 and LDHA, which enhances glucose uptake and lactate production, contributing to the pro-tumorigenic function of immunosuppressive cells such as TAMs, MDSC, and Treg cells. KRAS-induced high CIB1 expression regulates glycolytic metabolism, restricts CD8^+^PD-1^-^T infiltration and suppresses immune responses.

Oncogenic *KRAS* significantly impacts metabolism progresses across various pathways, with the regulation of glucose metabolism intermediates being dominant in most cancers. For instance, *KRAS*^G12D^ stimulates glucose metabolism to mediate tumor cell proliferation by inducing glucose intermediates into the hexosamine biosynthetic pathway and the PPP. *KRAS*^G12D^ transcriptionally regulates the glucose transporter and several rate-limiting enzymes, which collectively redirect glucose metabolism towards anabolic pathways [105]. *KRAS*^G12D^ was associated with the production of glycolytic intermediates, including glucose-6-phosphate (G6P), fructose-6-phosphate (F6P), and fructose-1,6-bisphosphate (FBP). *KRAS*^G12D^ reduction also down-regulated several rate-limiting glycolytic enzymes, such as hexokinase 1/2(HK1/2) and lactate dehydrogenase A (LDHA), which reduces glucose uptake and lactate production [105]. Notably, KRAS acts as a trigger to drive glucose metabolism, which promotes tumor proliferation. Sequentially, glucose accumulation in the TME induces genome destabilization and novel mutations, including additional the *KRAS* mutations [17]. *KRAS*-mutant tumor cells induce activation-induced cell death (AICD) of tumor-specific cytotoxic CD8^+^T cells by inhibiting the NF-κB pathway [106]. Mechanistically, lactate-derived histone lactylation produced by *KRAS* mutant tumor cells directly activates the transcription of circATXN7, an NF-κB-interacting circular RNA. CircATXN7 binds to the p65 subunit of NF-κB and blocks the nuclear localization signal motif of p65 in the cytoplasm, thereby enhancing the sensitivity of tumor-specific cytotoxic T cells to AICD [107]. KRAS-induced high calcium and integrin-binding protein 1 (CIB1) expression also alters the metabolic profile, leading to increased glycolysis metabolism, oxidative phosphorylation, and hypoxia pathway activation, which promotes tumor development (Fig. 5). CIB1 acts as a reprogrammed glucose metabolism mediator, restricting CD8^+^PD-1^−^T infiltration and suppressing the immune response in *KRAS*-mutant PDAC [19].

## Amino acids metabolism in the *KRAS*-mutant TME

**Fig. 6:**
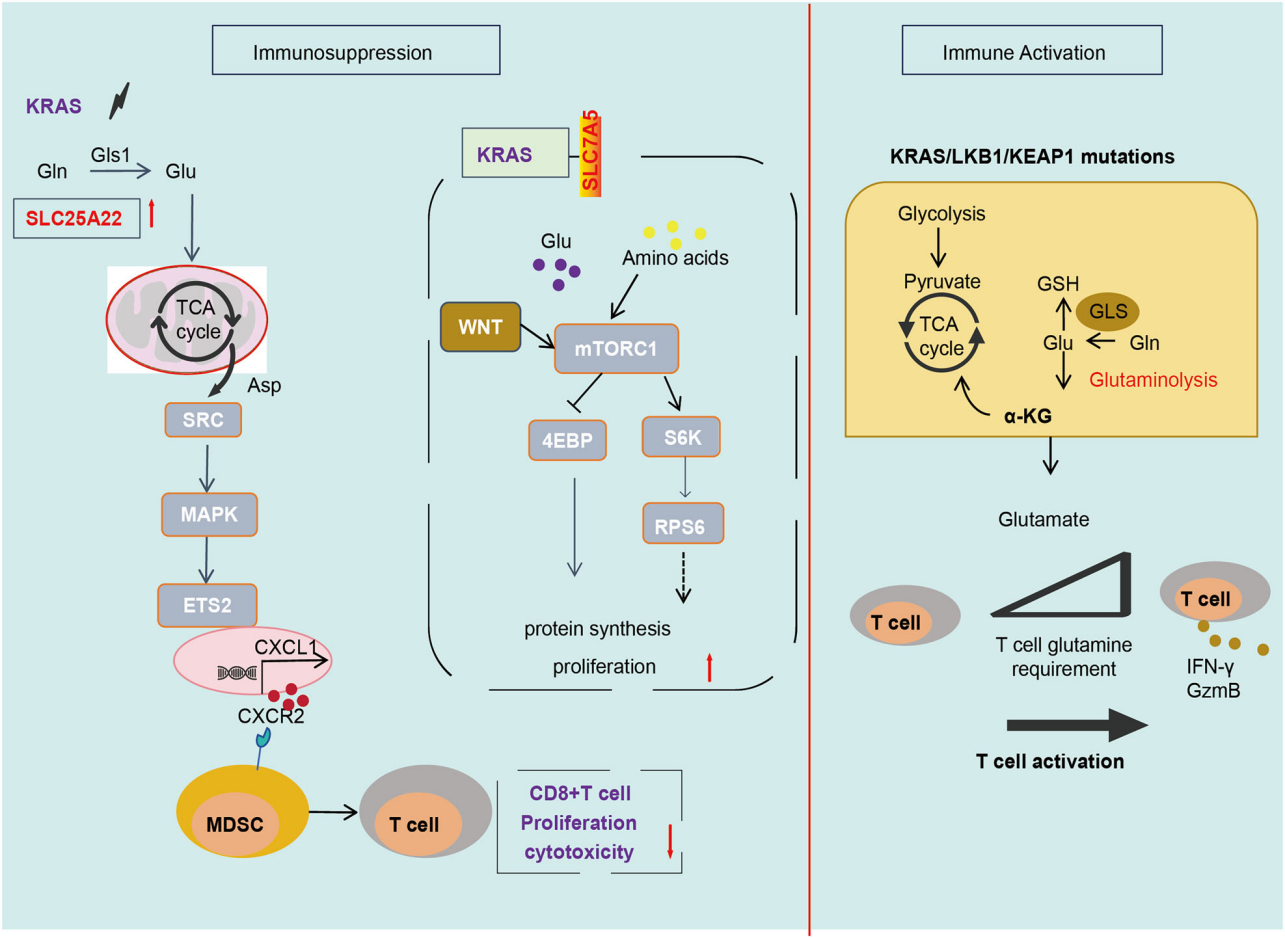
KRAS affects the TME by modulating amino acid metabolism. Mutant KRAS upregulates SLC25A22 expression, promotes ERK/ETS2 signaling, and activates CXCL1/CXCR2 pathway to recruit MDSC. KRAS regulates the SLC7A5/mTORC1 pathway to promote tumor proliferation. KRAS/LKB1 co-mutations increased glutamate levels and glutaminase expression. Glutamate abundance in LKB1-deficient TME promoted T cell activation in response to anti-PD-1.

Amino acids and their derivatives serve as important metabolic substrates that support *KRAS*-mutant cancer progression. In carcinoma cells, glutamine, an anaerobic metabolite, functions as a major amino acid that drives the TCA cycle to support the production of mitochondrial adenosine triphosphate. A coordinated metabolic network of glutamine transporters, glutaminases, transaminases, and redox homeostasis associated with glutamine metabolism is essential for cancer cell survival [108]. The reprogramming of glutamine metabolism is an important metabolic hub for immunoregulation in the TME. Oncogenic *KRAS* modulates intracellular amino acid levels through KRAS-dependent amino acid transporter expression and uptake. KRAS has been identified to participate in the immune response through amino acid metabolism, especially glutamine metabolism. Mutant *KRAS*^G12D^ promotes glutaminolysis via solute carrier family 25 member 22 (SLC25A22) as a member of the mitochondrial transporter family (SLC25) that facilitates glutamate metabolism [109]. In *KRAS*^G12D^-mutant CRC, SLC25A22 drives DNA methylation, activates oncogenic signaling, and enhances stem cell properties and resistance to treatment by promoting succinate accumulation, creating a positive feedback loop of cancer promotion [110]. SLC25A22 is a key molecule mediating metabolic reprogramming and immunosuppression in *KRAS* mutant CRCs. SLC25A22 could promote asparagine binding and activate SRC phosphorylation, and asparagine-mediated SRC facilitated ERK/ETS2 signaling, driving CXCL1 expression. Activation of the CXCL1/CXCR2 pathway recruited MDSCs, contributing to an immunosuppressive microenvironment [109]. The glutamine transporter SLC7A5 maintained intracellular amino acid levels through metabolic reprogramming upon KRAS activation. SLC7A5 was shown to induce protein synthesis required for tumor proliferation through mTORC1 [111]. In *KRAS*-mutant lung AD, LKB1 loss accompanied by KEAP1 mutations (KLK subtype), defined a distinct molecular subgroup. KLK tumors exhibit significant features of glutamine metabolism, TCA cycle, and redox homeostasis [112]. KRAS/LKB1 co-mutations alter the metabolic profile of tumors by increasing glutamate levels and glutaminase expression. Glutamate abundance in the LKB1-deficient TME facilitated the activation of T cells in response to anti-PD-1 [20], as illustrated in Fig. 6 and Table 2.

**Table 2:** KRAS and metabolism in TME.

Cancer type	Target	Metabolism type	Immune-related molecules	immune cells	Refs.
PDAC	UGCG	Glycosphingolipids	IFNγ /IFNGR1	NK and CD8 + T cells	[93]
Lung cancer	FASN	Phosphatidylcholines and triacylglycerides	—	—	[94]
PDAC	PPARδ	Lipid metabolism	CCL2/CCR2	Macrophages and MDSCs	[97]
CRC	GGPP	Cholesterol metabolism	IL-10	B cell	[98]
PDAC	CIB1	Glycolysis metabolism	PD-1	CD8 + T cells	[19]
PDAC	HK1/2 and LDHA	Lactate production	—	Macrophages	[105]
CRC	SLC25A22	Glutamate metabolism	CXCL1/CXCR2	MDSC	[109]
Lung cancer	SLC7A5	Glutamine metabolism	mTORC1	—	[111]

*PDAC*, pancreatic ductal adenocarcinoma, *UGCG* UDP-glucose ceramide glucosyltransferase, *IFNγ* interferon-gamma, *IFNGR1* interferon gamma receptor 1, *NK* natural killer cell, *FASN* fatty acid synthase, *PPARδ* peroxisome proliferator-activated receptor delta, *CCL2* C-C motif chemokine, *CCR2* C-C motif receptor 2, *MDSCs* myeloid-derived suppressor cells, *CRC* colorectal cancer, *GGPP* geranylgeranyl pyrophosphate, *IL-10* interleukin-10, *CIB1* chorionic gonadotropin beta polypeptide 1, *PD-1* programmed cell death protein 1, *HK1/2* hexokinase 1/2, *LDHA* lactate dehydrogenase A, *SLC25A22* mitochondrial glutamate carrier 1, *CXCL1* C-X-C motif ligand 1, *CXCR2* C-X-C motif chemokine receptor 2, *SLC7A5* solute carrier family 7 member 5, *mTORC1* mammalian target of rapamycin complex 1.

## KRAS targeted therapy and immunomodulatory-related clinical studies

KRAS mutation has long been considered “undruggable” owing to its unique spatial structural features. However, recent technological advancements have identified a “hidden pocket” of *KRAS*^G12C^ mutants in the KRAS structure that can be targeted. Consequently, the Lumakras (Sotorasib, AMG510) emerged as the first FDA-approved inhibitor for the treatment of patients with advanced NSCLC harboring the *KRAS*^G12C^ mutation [113]. Krazati (Adagrasib) was approved as the second inhibitor indicated for patients with locally advanced or metastatic NSCLC harboring the *KRAS*^G12C^ mutation [114]. In addition to *KRAS*^G12C^ mutations, the *KRAS*^G12D^ mutation has been frequently detected among patients. Current evidence suggests that MRTX1133 selectively binds to *KRAS*^G12D^ mutants and can inhibit KRAS-dependent related signaling pathways [115]. The pan-KRAS inhibitors (e.g., RMC-6236, YL-17231, JAB-23425, JAB-23400) mainly targeted to Son of Sevenless 1 (SOS1) proteins, which inhibited their capacity to catalyze the binding of KRAS to GTP, as well as the transition of KRAS from inactive to active state, inactivating KRAS, and consequently inhibiting tumor development [116, 117].

Despite the large number of RAS inhibitors being tested in clinical trials, resistance-related issues have gradually emerged in individual patients treated with *KRAS*^G12C^ inhibitors [118]. Preclinical evaluation found that sotorasib alone caused short-term tumor reduction followed by tumor regrowth in a immunocompetent mouse CRC cell line-derived xenograft with *KRAS*^G12C^-mutant. When used in combination with anti-PD-L1 therapy, complete tumor regression was detected, persisting for more than 2 months after treatment ended [119]. Researchers have attempted to develop inhibitor combinations as immunotherapies targeting mutation-specific immune microenvironments of tumors with *KRAS* mutations to further improve patient outcomes [120, 121]. Mechanistic studies revealed that AMG510 fostered a pro-inflammatory TME, increases T cell infiltration, and exhibited synergistic effects with ICB therapy [119]. MRTX1133 was found to significantly modulate myeloid cell subsets or macrophage function, inhibiting the differentiation of Arg1^+^macrophages and promoting the infiltration of Mrc1^+^macrophage populations. Adagrasib treatment upregulated the expression of MHC class I, reduced the levels of immunosuppressive factors, decreased the abundance of myeloid-derived suppressor cells and increased the number of M1-polarized macrophages, dendritic cells, CD4^+^, and CD8^+^ T cells [122]. In addition, MRTX1133 regulated interactions between cancer cells and CD8^+^T cells by FAS-FASL signaling, promoting the cytotoxic activity of CD8^+^T cells, and synergized with ICI to remodel the TME [13]. The HRS-4642 specifically bound to *KRAS*^G12D^ and inhibited the phosphorylation of MEK/ERK, thereby suppressing tumor progression [123, 124]. HRS-4642 treatment increased the proportion of CD45^+^, CD4^+^, and CD8^+^ leukocytes and the number of effector T cell populations (CD44^+^CD62L^−^ CD4^+^ or CD8^+^ cells) [123].

Numerous clinical trials have evaluated the efficacy of KRAS inhibitors combined with anti-PD-1/L1 in the treatment of patients with advanced solid tumors (Clinical Trials.gov identifier: NCT04185883, NCT03785249). In this respect, the LOXO-RAS-20001 study found that Olomorasib (LY3537982) may serve as a second-generation KRAS^G12C^ inhibitor, exhibited good efficacy when combined with pabolizumab. Among 43 patients with NSCLC, with 81% (*n* = 35/43) receiving prior immunotherapy, the objective response rate (ORR) was 40% (*n* = 17/43). In the first-line metastatic (*n* = 17) group, the ORR was 77%, with a disease control rate (DCR) of 88% [125, 126]. In the KRYSTAL-7 trial, the efficacy and safety of KRAS^G12C^ inhibitor adagrasib plus pembrolizumab were examined in patients with treatment-naïve, advanced NSCLC harboring *KRAS*^G12C^ mutations. It was observed that, among the 51 patients with PD-L1⩾50%, the ORR was 63% (32/51, 95% CI: 48%–76%) with a DCR of 84% (43/51; 95% CI: 71–93). However, mPFS and DOR were not reached within a median follow-up of 10.1 months [127]. Collectively, these data demonstrate that the combinations of KRAS^G12C^ inhibitors and immunotherapy may yield good clinical efficacy (Table 3). Further clinical studies are needed to validate these findings.

**Table 3:** Clinical trials evaluating the combination of KRAS inhibitors with immune checkpoint inhibitors.

Drug	Combination	Cancer Type	Study overview	Clinical trial
Sotorasib	Pembrolizumab (PD-1)	mCRC、NSCLC	Phase 1: safety and tolerability	CodeBreak101 (NCT04185883)
	Atezolizumab (PD-L1)		Phase 2: ORR、DCR、DoR	
Adagrasib	Pembrolizumab (PD-1)	NSCLC	Phase 1: safety and tolerability	KRYSTAL-1 (NCT03785249)
			Phase 2: ORR、DCR、DoR	
LY3537982	Pembrolizumab (PD-1)	NSCLC	Phase 1: safety and tolerability	LOXO-RAS-20001(NCT04956640)
			Phase 2: ORR、DCR DoR	
JDQ443	Tislelizumab (PD-1)-/+	Solid tumor	Phase 1/2: safety, tolerability, and tumor activity	KontRASt-01 (NCT04699188)
	TNO155(SHP2i)			
Adagrasib	Pembrolizumab (PD-1)	NSCLC	Phase 2: safety and tolerability of monotherapy and combination in advanced NSCLC and any PD-L1 TPS candidates for first-line treatment Phase 3: compare efficacy of adagrasib plus pembrolizumab vs. pembrolizumabin first-line setting with tumors PD-L1 TPS ≥ 50%	KRYSTAL-7 (NCT04613596)
GDC-6036	Atezolizumab (PD-L1)		Phase 1: safety and tolerability	NCT04449874
			Phase 2: ORR、PFS	
MK-1084	Pembrolizumab (PD-1)	NSCLC	Phase 1: safety and tolerability	NCT05067283
MK-1084	Pembrolizumab (PD-1)	NSCLC (*n* = 600) PD-L1 TPS ≥ 50%	Phase 1: safety and tolerability	NCT06345729
RMC-6291(G12C)or RMC-6236(multi) Revolution Medicines	Pembrolizumab (PD-1) −/+chemotherapy	NSCLC	Phase 1/2: safety, tolerability, and tumor activity combined with standard of care (PD-1-/+chemo)	NCT06162221
IBI351	Sintilimab (PD-1)−/+ chemotherapy	NSCLC	Phase 1: safety and tolerability of monotherapy and combination in NSCLC Phase 2/3: efficacy	NCT05504278

*ORR* objective response rate, *DCR* disease control rate, *DOR* duration of response, *PFS* progression-free survival, *TPS* tumor proportion score.

Besides PD-1/L1 single-agent immunotherapy, other immunomodulatory treatments for *KRAS*-mutant cancers are undergoing clinical trials. It has been reported that the IL-1β monoclonal antibody, canakinumab significantly reduces lung cancer incidence and mortality by inhibiting inflammation in *KRAS*-mutant NSCLC [128]. Moreover, the anti-IL-6 monoclonal antibody saltuximab showed marginal benefits when applied as monotherapy in *KRAS*-mutant solid tumors (NCT00841191) [129]. Neoantigens generated by mutations in tumor DNA are among the targets for developing tumor immunotherapy. A Phase Ib clinical trial found that neoantigen-vaccine NEO-PV-01 reshapes the TME of *KRAS*^G12C^-mutated NSCLC, including activated T cell cytotoxicity, enhanced tetramer^+^ (neoantigen-specific) CD4^+^ T cell populations, high expression of MHC class II by cells of the monocytic lineage (CD14, CD11c), and increased TCR diversity (Clinical Trials.gov identifier: NCT03380871) [130]. Other clinical studies have demonstrated that SHP2-based combination therapy could effectively control the progression of *KRAS*-mutant solid tumors.

It has been established that SHP2 is required for activating RAS, enabling SHP2i to block downstream signaling triggered by overactive receptor tyrosine kinases (RTKs) and RAS mutants. As a protein expressed in both tumor and immune cells, SHP2 exhibited dual functions: in tumor cells, SHP2 promotes tumor formation and progression via the RTK-RAS-ERK signaling pathway. In immune cells, SHP2 can dampen CD8^+^T cell cytotoxicity and macrophage phagocytosis. The SH2 domain of SHP2 can interact with the immunoreceptor tyrosine-based switch motif (ITSM) of PD-1 at the Y248, activating SHP2 and mediating the inhibitory function of PD-1 [131]. Several clinical trials have investigated the efficacy of SHP2 inhibitor-based combination immunotherapy or KRAS-targeted Sotorasib in patients with *KRAS* mutations, reporting positive results. These studies collectively demonstrate that SHP2 inhibitors are potential drugs for cancer immunotherapy (ClinicalTrials.gov identifier: NCT05375084, NCT05480865). However, future studies are warranted to develop targeted therapies or combination therapies leveraging the immune microenvironment characteristics and metabolic targets in KRAS-driven cancers.

## Conclusions

The *KRAS*-mutant tumors exhibit mutation-specific TMEs, primarily accounting for the heterogeneity of anti-tumor responses of immunotherapy in *KRAS*-mutant tumors. The present review expounded on the diverse immune-related molecules and inflammatory factors regulated by KRAS which irreversibly affect the TME and the direct effects of KRAS-downstream signaling on tumorigenesis. The reviewed data indicate that activating *KRAS* mutations exert diverse effects on immune cells by modulating immune-related molecules, such as macrophage polarization and function, enhancing the differentiation of T cells to Treg-like cells, inhibiting T cytotoxicity, and accelerating T-cell exhaustion. Moreover, KRAS remodels the immune microenvironment by influencing tumor metabolism, an important route for immunomodulation. Metabolite accumulation in the TME induces genome destabilization and the occurrence of mutations, such as the *KRAS* mutation.

KRAS activation regulates the expression of immune-related molecules, inflammatory signals, and metabolic pathways through direct or indirect cascading mechanisms, creating interdependent relationships. The aberrant expression of immune-related molecules establishes an “immune escape foundation”. The sustained release of inflammatory factors serves as the “driving force” for metabolic abnormalities. Meanwhile, the accumulation and exploitation of metabolic pathways offer the “microenvironmental support” for immune suppression and the maintenance of inflammation, which ultimately work synergically to drive the tumor immune microenvironment into a suppressive state in KRAS-mutant tumors. Remodeling immune cell characteristics and functions represents a promising approach to reshape the immune microenvironment of *KRAS*-mutant tumors. Therapeutic interventions targeting immune-related molecules and metabolism in *KRAS*-mutant tumors have huge potential, which need to be tested in future clinical studies.
